# Draft Genome Sequence of Caprolactam-Degrading *Pseudomonas putida* Strain SJ3

**DOI:** 10.1128/genomeA.00810-15

**Published:** 2015-07-23

**Authors:** Sung-Jun Hong, Gun-Seok Park, Abdur Rahim Khan, Byung Kown Jung, Yeong-Jun Park, Na-Kyung Yoo, Changhee Lee, Choi Kyu Park, Jae-Ho Shin

**Affiliations:** a School of Applied Biosciences, Kyungpook National University, Daegu, Republic of Korea; b Product Development & Customer Care Division, Celemics Inc., Seoul, Republic of Korea; c School of Life Sciences and Biotechnology, College of Natural Sciences, Kyungpook National University, Daegu, Republic of Korea; d College of Veterinary Medicine, Kyungpook National University, Daegu, Republic of Korea

## Abstract

*Pseudomonas putida* strain SJ3, which possesses caprolactam-degrading ability, was isolated from dyeing industry wastewater in Daegu, Republic of Korea. Here, we describe the draft genome sequence and annotation of the strain. The 5,596,765-bp-long genome contains 4,293 protein-coding genes and 68 RNA genes with 61.70% G+C content.

## GENOME ANNOUNCEMENT

Most of the *Pseudomonas putida* strains that have been isolated and characterized so far are usually found as innocuous environmental microorganisms with a great potential for biotechnological applications because of their metabolic versatility and adaptability (1). *P. putida* strains are metabolically versatile and thrive in diverse habitats. Strains of this species are known for their ability to colonize soil and strongly participate in bioremediation and degradation of a wide variety of chemicals, including natural and man-made compounds such as ɛ-caprolactam, naphthalene, and toluene (2–4). ε-caprolactam is synthesized synthetically from benzene and used almost exclusively for nylon-6 production. Nylon-6, a man-made polymer, has wide applications in the manufacturing of fabrics, automobiles parts, car tires, ropes, etc. (5). Because of its mass production, vast quantities of wastewater are generated during the manufacturing process, which is mostly discharged into natural water reservoirs without treatment (6).

To isolate caprolactam-degrading microorganisms, wastewater samples were spread on NB agar plates containing 50 mM caprolactam. From the initial caprolactam-tolerant screening, seven isolates showed varying degrees of tolerance. Subsequently, these strains were further screened for caprolactam degradation potential, and strain SJ3 showed the best degradation rate.

Microbial whole-genome sequencing was done using an Ion Torrent personal genome machine with a 200-bp single-end library and a 5 kb mate pair library, which generated 2,685,794 fragment reads (61× coverage of genome) and 1,097,427 mate pair reads (55× coverage of genome). The fragment reads were assembled with Mira Assembler version 3.4.0 and CLC Genomics Workbench 6.0. Assembly produced 424 contigs (400 bp or more), with an *N*_50_ contig length of 19,018 bp. For mate pair data, DNAStar version 11.0 was applied and generated 279 final contigs and an *N*_50_ contig length of 32,019 bp. The draft genome sequence consisted of 5,596,765 nucleotides with 61.70% G+C content. Subsequent to the assembly, the contigs were submitted to the RAST annotation server (http://rast.nmpdr.org/) for subsystem classification and functional annotation (7). The annotation results revealed 4,293 predicted coding sequences, including 60 tRNAs, 5 rRNAs, and 3 noncoding RNAs.

We looked for genes involved in caprolactam degradation using next-generation sequencing (NGS) as in previous studies (8, 9). Polycyclic aromatic hydrocarbons (PAHs) and dioxin-like compounds have been widely identified in the environment and in industrial production waste (10). Many of these contaminated habitats are also characterized by high concentrations of organic solvents (11). The versatile metabolic capacity of the pseudomonads enables efficient degradation of these compounds (12, 13). Several of the genes found in strain SJ3 were mapped to the degradation pathways of aminobenzoate, benzonate, bisphenol, and dioxin within the KEGG orthology database, similar to previous NGS studies. The genome sequencing of this strain will provide great insight into its genetic variability and the biodegradation of a diverse range of chemical compounds. In a future study, we will investigate the application of degradation in strain SJ3 to environmental contamination.

### Nucleotide sequence accession numbers.

This whole-genome shotgun project has been deposited at DDBJ/EMBL/GenBank under the accession number AXDX00000000. This version of the second project is AXDX02000000. The 279 contigs have been deposited under the accession numbers AXDX02000001 to AXDX02000279.

## References

[1] Timmis KN 2002 *Pseudomonas putida*: a cosmopolitan opportunist par excellence. Environ Microbiol 4:779–781. doi:10.1046/j.1462-2920.2002.00365.x. 12534460

[2] Zylstra GJ, McCombie WR, Gibson DT, Finette BA 1988 Toluene degradation by *Pseudomonas putida* F1: genetic organization of the tod operon. Appl Environ Microbiol 54:1498–1503. 2843094 10.1128/aem.54.6.1498-1503.1988 PMC202686

[3] Gomes NC, Kosheleva IA, Abraham W-R, Smalla K 2005 Effects of the inoculant strain *Pseudomonas putida* KT2442 (pNF142) and of naphthalene contamination on the soil bacterial community. FEMS Microbiol Ecol 54:21–33. doi:10.1016/j.femsec.2005.02.005. 16329969

[4] Marqués S, Ramos JL 1993 Transcriptional control of the *Pseudomonas putida* TOL plasmid catabolic pathways. Mol Microbiol 9:923–929. doi:10.1111/j.1365-2958.1993.tb01222.x. 7934920

[5] Szostak-Kotowa J 2004 Biodeterioration of textiles. Int Biodeterior Biodegradation 53:165–170. doi:10.1016/S0964-8305(03)00090-8.

[6] Baxi NN 2013 Influence of ε-caprolactam on growth and physiology of environmental bacteria. Ann Microbiol 63:1471–1476. doi:10.1007/s13213-013-0610-4.

[7] Aziz RK, Bartels D, Best AA, DeJongh M, Disz T, Edwards RA, Formsma K, Gerdes S, Glass EM, Kubal M, Meyer F, Olsen GJ, Olson R, Osterman AL, Overbeek RA, McNeil LK, Paarmann D, Paczian T, Parrello B, Pusch GD, Reich C, Stevens R, Vassieva O, Vonstein V, Wilke A, Zagnitko O 2008 The RAST server: Rapid Annotations using Subsystems Technology. BMC Genomics 9:75. doi:10.1186/1471-2164-9-75. 18261238 PMC2265698

[8] Buell CR, Joardar V, Lindeberg M, Selengut J, Paulsen IT, Gwinn ML, Dodson RJ, Deboy RT, Durkin AS, Kolonay JF, Madupu R, Daugherty S, Brinkac L, Beanan MJ, Haft DH, Nelson WC, Davidsen T, Zafar N, Zhou L, Liu J, Yuan Q, Khouri H, Fedorova N, Tran B, Russell D, Berry K, Utterback T, Van Aken SE, Feldblyum TV, D’Ascenzo M, Deng W-L, Ramos AR, Alfano JR, Cartinhour S, Chatterjee AK, Delaney TP, Lazarowitz SG, Martin GB, Schneider DJ, Tang X, Bender CL, White O, Fraser CM, Collmer A 2003 The complete genome sequence of the Arabidopsis and tomato pathogen *Pseudomonas syringae* pv. tomato DC3000. Proc Natl Acad Sci U S A 100:10181–10186. doi:10.1073/pnas.1731982100. 12928499 PMC193536

[9] Silby MW, Cerdeño-Tárraga AM, Vernikos GS, Giddens SR, Jackson RW, Preston GM, Zhang X-X, Moon CD, Gehrig SM, Godfrey SA, Knight CG, Malone JG, Robinson Z, Spiers AJ, Harris S, Challis GL, Yaxley AM, Harris D, Seeger K, Murphy L, Rutter S, Squares R, Quail M, Saunders E, Mavromatis K, Brettin T, Bentley S, Hothersall J, Stephens E, Thomas C, Parkhill J, Levy S, Rainey P, Thomson N 2009 Genomic and genetic analyses of diversity and plant interactions of *Pseudomonas fluorescens*. Genome Biol 10:R51. doi:10.1186/gb-2009-10-5-r51. 19432983 PMC2718517

[10] Xu P, Yu B, Li FL, Cai XF, Ma CQ 2006 Microbial degradation of sulfur, nitrogen and oxygen heterocycles. Trends Microbiol 14:398–405. doi:10.1016/j.tim.2006.07.002. 16860985

[11] Sardessai YN, Bhosle S 2004 Industrial potential of organic solvent tolerant bacteria. Biotechnol Prog 20:655–660. doi:10.1021/bp0200595. 15176865

[12] Inoue A, Horikoshi K 1989 A Pseudomonas thrives in high concentrations of toluene. Nature 338:264–266. doi:10.1038/338264a0.

[13] Li Q, Wang X, Yin G, Gai Z, Tang H, Ma C, Deng Z, Xu P 2009 New metabolites in dibenzofuran cometabolic degradation by a biphenyl-cultivated *Pseudomonas putida* strain B6-2. Environ Sci Technol 43:8635–8642. doi:10.1021/es901991d. 20028064

